# Semantic Typicality of Affixes Facilitates Word Processing: MEG Evidence From Arabic

**DOI:** 10.1162/NOL.a.24

**Published:** 2025-12-01

**Authors:** Marianne Azar, Alec Marantz

**Affiliations:** Department of Psychology, New York University, New York, NY, USA; Department of Linguistics, New York University, New York, NY, USA

**Keywords:** affixes, Arabic, MEG, morphology, semantics

## Abstract

Upon reading a word, we decompose it into meaningful parts—morphemes. Even if novel, we can derive a likely meaning for it based on how its parts typically behave. Given the typical meaning of affix “-ery,” we may guess that a bottlery is a place to make bottles, although we may alternatively guess that it is the craft of bottle making. In this study, we operationalize this feature—an affix’s semantic typicality—to investigate affix semantics’ role in word processing. Using a lexical decision task and a double dissociative design in an MEG setting, we took advantage of Arabic’s highly productive word-pattern derivational system to investigate the role of meaning typicality for derivational morphology. We contrasted one affix typically denoting tools and atypically places with another affix having the reverse denotation pattern. We found higher activity for typical-meaning words in the temporal pole, inferior temporal gyrus, and middle temporal gyrus at an earlier time window than previously associated with semantic processing. Additionally, we replicated results on noun/verb ambiguity, where ambiguous words had higher activity in the fusiform gyrus and throughout the temporal lobe. Our results on lexicality—contrasting words versus nonwords—were also consistent with previous literature. A finer-grained distinction between pseudowords with real roots versus pseudoroots further allowed us to explore the role of affixes in processing in the temporal pole and the inferior frontal cortex. Overall, our study contributes importantly to findings on affix semantic processing and contributes generally to growing findings on different stages of morphological decomposition.

## INTRODUCTION

When we encounter a word, we activate related words in our mind. This association may be based on meaning or on form. Meaning-based associates of “happiness” could be “smile,” “laugh,” “good.” Based on form, they may be “happy,” “unhappy,” “happily.” Importantly, “happiness” is made of two morphemes, “happy” and “-ness,” and its form-based association may be based on either morpheme: “happy,” “unhappy,” “happily”; or “tiredness,” “illness,” “lightness.” Very often, shared form (here, a morpheme) denotes shared meaning. In this example, the shared meaning is either related to the root “happy,” or the meaning of a state, which the affix “-ness” denotes.

In the neuroscientific study of form and meaning—morphosemantics—much attention has been given to roots as words’ primary locus of meaning, despite the intuitive meaningfulness of affixes such as “-ness,” “-ery,” “-ist.” The semantic contribution of affixes has largely been backgrounded, and when it has been studied, it has been treated as secondary to the root meaning in determining (or only modifying) word meaning. In the languages studied most in psycholinguistics, roots are mostly free morphemes, able to occur by themselves as words, whereas affixes are bound morphemes that must be attached to a root. This bias in languages studied may motivate the perceived primary role of roots. For example, the standard measure of a word’s compositional *opacity* compares the meaning of a root (“happy”) with the meaning of the derived form (“happiness”), but does not assess the opacity of “-ness,” by comparing “happiness” and “illness,” for instance. Asking speakers to judge the closeness of meaning between a root and a derived form necessitates that the root stands alone as an interpretable word. What happens then in a language where roots cannot stand alone?

To study the role of affixes, an important distinction must be made between roots and free morphemes. The independence of these classes is clearly illustrated in Arabic, where roots and affixes are both bound morphemes with indeterminate meanings, both of which need to be present to form a word. An example is illustrated in Table 1.

**Table 1.** Root-and-pattern Arabic word composition

Triconsonantal root (thematic lexical field)	Pattern (taxonomic lexical field)			Word	
Vague semantic meaning. Underspecified grammatical category	Vague semantic meaning. Provides grammatical category			Specific meaning, unambiguous or disambiguated by phrase context	
				Taxonomic relation	
				Place	Person
KTB (*writing*)	maCCaC; Typically, a place. Less typically a concept, thing or event. Nominal	Thematic relation	Writing (KTB)	Ma**kt**a**b** (*Office: writing place*)	**K**a:**t**e**b** (*Writer: writing person*)
LJʔ (*seeking safety*)	Ca:CeC; Typically, an agent. Also, an active participle. Nominal or verbal		Seeking safety	Ma**lj**a**ʔ** (*Shelter: Seeking-safety place*)	**L**a:**j**e**ʔ** (*Refugee: Seeking-safety person*)

*Note*. The root is formed of three consonants (CCC) and combines with a pattern to form a fully specified word. Both root and pattern individually provide semantic meaning, and the pattern also provides grammatical information.

This feature of Arabic (and other Semitic languages) provides an excellent opportunity to study the semantic contribution of affixes and their processing within a word. Critically, this processing must reflect that often one affix can mean different things. For example, in English, most “-ery” suffixed words are places, though the family less commonly denotes concepts (“savagery”), or collections (“cutlery”). In Arabic as well, one affix, for instance, can mostly denote places, but less frequently tools or concepts. We hypothesize that if the semantic contribution of affixes plays a role in word processing, then the neural signature of processing words with a meaning consistent with a common denotation of their affix (*affix-typical words*), will be distinct from the neural signature of the processing of their counterpart (*affix-atypical words*). Drawing on previous findings on morphological decomposition, recombination, and semantic coherence, we can predict different typicality effects at different stages of processing, which we will discuss below. To test this hypothesis, we chose a double dissociation between two affixes and typicality/atypicality of meaning in a 2 × 2 design. This design, alongside the similar role of affixes and roots in word formation in Arabic, provides an excellent window to examine the semantic contribution of affixes in word comprehension, and gives a more comprehensive account of the neural mechanisms supporting word comprehension across languages.

### Affix Meaning Through Two Lenses: Secondary or Comparable to Root

In the literature investigating word meaning, a distinction emerges in relation to affix meaning within a word. Affixes are operationally defined either as secondary to roots in word meaning (modulating root meaning) or as being comparable to roots in denoting word meaning, although this last definition is more present in the theoretical literature than the empirical literature.

#### Affix as secondary to root

Defining affixes as secondary to roots in semantic contribution assesses word meanings with their root providing base meaning and an affix modulating this meaning. The result varies in its semantic distance from the simple word (close: “casual”-“casualness”; distant: “casual”-“casualty”). Critically, this definition has been introduced and assessed through languages where the root is often a free morpheme. If the root can be assigned a meaning without an additional morpheme, speakers (and language models) *can* judge the semantic similarity between the root and the derived form, yielding a measure of the opacity of the combination of root and suffix. This methodology, though, conflates free morphemes with roots. Consider bound roots, such as “amen-“ in “amenity,” “amenable,” which has an polysemous or vague (if any) meaning before its combination (see Jackendoff & Audring, 2020, for a discussion). Then consider Arabic, where all roots are bound, are syntactically underspecified and semantically vague, as illustrated in Table 1. With these examples, the notion of a root being an independent locus of meaning does not stand up to scrutiny. Moreover, all roots, whether free or bound, have been found to decompose in early stages of complex word processing (Lewis et al., 2011).

Previous relevant morphosemantic studies have largely treated affixes as modulating root meaning: for example, an fMRI study of semantic transparency (whether related to stem or not) and suffix productivity in Italian (Carota et al., 2016); and a behavioral priming study of German prefixed versus particled verbs (Smolka et al., 2019). A magnetoencephalography (MEG) priming lexical decision study by Cavalli et al. (2016) studying French nominals had important findings for the neural temporal dynamics of morphological and semantic processing. Similarly to Smolka et al. (2019), complex word primes, which primed their root words as targets, were manipulated within the design to separate morphosemantic and morpho-orthographic effects. We outline their design and findings in Table 2.

**Table 2.** Summary of Cavalli et al. (2016) MEG priming lexical decision study experimental design and findings

Experimental design			
Contrast			Example: prime-TARGET; translation
+ morphology + semantics			ourson-OURS; cub-BEAR
+ semantics			peluche-OURS; plushie-BEAR
+morphology +orthography			oursin-OURS; urchin-BEAR
unrelated			gésier-OURS; gizzard-BEAR
Findings			
ROI	Time	Effect	Related findings
Left FFG	100 ms	orthographic	Tarkiainen et al., 1999; Gwilliams et al., 2016
Left STG	585–650 ms	morphosemantic, not semantic	Fruchter & Marantz, 2015; Neophytou et al., 2018
Left IFG	350–460 ms	morpho-orthographic	Morphological: Bozic et al., 2007; Davis et al., 2004 Semantic processing: Price, 2012 Lexical orthographic processing: Montant et al., 2011
	440–495 ms	morphosemantic	
Left OFC	435–500 ms	morphosemantic	Fruchter & Marantz, 2015

*Source*: Cavalli et al. (2016). ROI = region of interest; FFG = fusiform gyrus; STG = superior temporal gyrus; IFG = inferior frontal gyrus; OFC = orbitofrontal cortex.

Altogether, these results support a hypothesis of a significant interdependence between morphological form and semantic processing. Importantly, however, the studies outlined in Table 2 did not manipulate affix meaning in their designs. Consequently, they rather address specifically root semantics and morphology.

#### Affix as comparable to root

Although the experimental literature in morphosemantics has largely not addressed the semantics of affixes, it has been discussed in the theoretical literature. Lieber (2004) describes the approach of seeing affixes as modifying meaning as “the lexical semantics of words” (p. 2) rather than “the lexical semantics of *word formation*” (p. 2). The former approach excludes the possibility that morphemes may be semantically underspecified while contributing to a fully specified meaning of a word. In the latter approach, however, each type of morpheme is viewed as making a different type of semantic contribution. This distinction becomes clearer when considering these semantic features of morphemes: the vagueness of bound roots (e.g., “leg-” in “legitimate,” “legible,” “legal or “nat-” in “native,” “natal,” “nation,” “nature”), the semantic shift between words within a root’s derivational family (e.g., “casual”-“casualty”), and distinct semantic categories present within affix derivational families. Given these, both roots and affixes may be polysemous to a degree and contribute differently to the full meaning specification of different words in which they occur.

The semantic shift over members of a family of words sharing a root is analogous to the semantic diversity within an affix’s family of words. Finally, this pattern is clearest in a language such as Arabic, where virtually all roots are bound and virtually all words are obligatorily formed of a root and derivational affix. Given these points, a conceptualization of the semantics of word formation must be able to accommodate a view of affixes not being secondary to roots in their contributions to a word’s meaning.

### Affix Meaning Typicality

What we will introduce as a factor to consider in processing is the informational content of affixes. Words of an affix derivational family can denote one of a few semantic categories contained within words of the family. Table 3 provides a contrast of same meanings with distinct affixes where the meaning is typical or atypical for those affixes (see Table S4B in the Supplementary Materials, available at https://doi.org/10.1162/NOL.a.24, for examples of typical and less typical meanings of words sharing an affix). Affixes have more or less typical meanings based on their relative family size. For instance, there are more place “-ery” words (typical) than collection “-ery” words (atypical). We define typicality as the reliability that a word-class defined by one feature (e.g., affix) shares another feature (e.g., semantic category). Affix semantic typicality, then, is defined as the property of an affix reliably producing a word of a certain semantic category. The modal semantic category denoted by an affix would be considered the typical meaning of that affix. If meaning typicality is relevant in processing affixes, then we should find a distinction in the neural signature associated with the processing of affix-typical and affix-atypical words, independent of other features such as stem or whole-word frequency.

**Table 3.**
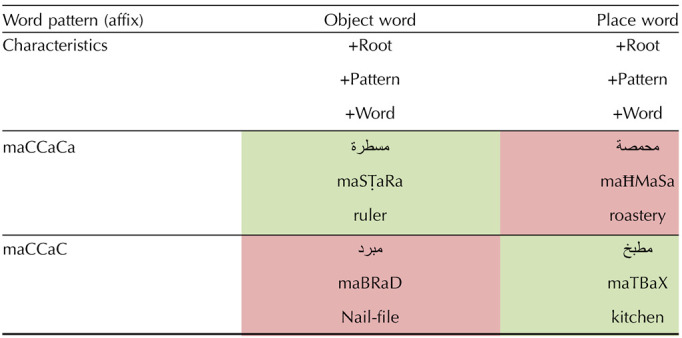
Semantic typicality manipulation design

*Note*. Cells in green: typical meaning-to-affix relation. Cells in red: atypical meaning-to-affix relation.

### Arabic Derivational Affixes: Necessary and Informative

In Arabic, words are realized as a (generally) triconsonantal root linked to consonant (C) positions in a consonant-vowel (CV) word pattern. Only a highly restricted set of words (120 primitive words) may be argued to not contain a pattern (nature words, e.g., “salt,” “sea,” “sand,” “mountain,” “earth”). Arabic word formation and the role of roots and affixes is illustrated in Table 1 (see examples for shared root words in Supplementary Table S4A and shared affix words in Supplementary Table S4B).

A series of behavioral studies have shown comparable results of priming between affixes and roots in Arabic (Boudelaa & Marslen-Wilson, 2015). Comparing the priming of Arabic affixes to suffix priming and Arabic underspecified roots to stems in other languages, affix priming was found to be significant in Arabic, whereas it has been inconsistently observed for suffixes in non-Arabic languages (no effect found in French in Meunier et al., 2000, nor in English in Marslen-Wilson et al., 1994; whereas, also in English, an effect of derivational suffix priming was found, even at long distances, by Gaston et al., 2021; in Hebrew, an effect was sound for verbal forms but not nominal forms by Deutsch et al., 1998).

In a series of cross-modal priming behavioral experiments assessing word patterns and roots in both nominal and verbal forms, Boudelaa and Marslen-Wilson (2015) provide strong support for the prevalent role of morphological decomposition in the processing of different complex words in Arabic across different morpheme types. The design and findings of the experiments are summarized in Table 4.

**Table 4.** Summary of design and results of Boudelaa and Marslen-Wilson (2015)

Target Conditions			1	2	3	4
Experiment	Prime-target syntax		+morphophonology +morphosemantic	+morphophonology −morphosemantic	Phonological control	Unrelated
Word patterns	1	nominal	✓	x	x	x
	2	verbal	✓	✓	x	x
					Semantic control	
Roots	3	nominal	✓	✓	x	x
	4	mismatch	✓	✓	x	x
			+root +semantic	+word pattern +semantic	Phonological control	
Primitive nouns	5	nominal	✓	x	x	x

Morphological priming was found robustly both with roots and word patterning (with a mismatch of effects between verbal and nominal patterns sharing form but not meaning, possibly due to a greater perceived difference between the nominal forms (e.g., plural vs. state) versus verbal forms (causative vs. intensive). In experiment 5, root priming was further found even with words with very low productivity roots (120 words in all the language). Word-pattern priming was absent, however, suggesting that it is dependent on root productivity. Given the prevalent interdependence of roots and affixes in Arabic and their demonstrated comparable role in word processing, Arabic offers an excellent opportunity to investigate affix semantic processing.

### Processing of Visually Presented Words

Below we will present the relevance of different affix variables throughout the temporal and spatial trajectory of single word visual word processing in the brain, basing our predictions on previous findings related to the processing of form and meaning, and relating them to the properties of Arabic affixes, semantic typicality, syntactic processing, and lexicality.

#### Early processing: Decomposition

Different theories make contrasting claims on the processing of morphologically complex visually presented words. The full listing model (Butterworth, 1983) predicts that all words are accessed and stored in the mental lexicon as whole words. In stark contrast, the full decomposition model (Rastle & Davis, 2008; Stockall & Marantz, 2006; Taft, 2004; Taft & Forster, 1975) predicts that all words are decomposed into their constituent morphemes during recognition (e.g., “unhappily”: “un-” + “happy” + “-ly”), with individual morphemes stored independently in the mental lexicon. Given that this latter model has been substantially supported across methodologies (see Rastle & Davis, 2008, for a review), we are adopting its assumptions in this study.

MEG studies have found reliable early morphological decomposition effects, with more activity for multimorphemic words of different types bilaterally, marked by an effect in the visual word form area (VWFA) at 130–210 ms (Zweig & Pylkkänen, 2009). Additionally, the frequency of morpheme combinations (the transition probability from stem to affix) has been shown to modulate activity in the VWFA (Lewis et al., 2011; Solomyak & Marantz, 2010) at around 170 ms post-stimulus onset. This is known as the M170 effect—an MEG response peak between 150–200 ms post-word onset characteristically in the inferior occipitotemporal area (specifically, fusiform area) that is sensitive to morphological complexity of words in (at least) the left hemisphere. Concerning our study, if a larger M170 amplitude is observed for typical affix uses, it points to top-down long-term effects on the ortho-morphemic processing of affix-root combinations, with typical words being a sharper combination of affix and root.

Importantly for the case of Arabic nonconcatenative root affix combinations, morphological decomposition has also been found for irregular verbs in English (Fruchter et al., 2013), indicating that decomposition does not depend on the linearity or regularity of morpheme combinations, in contrast to the predictions of a dual mechanism rules-and-lists model (Pinker & Prince, 1988), and implies that decomposition effects will be replicated in Arabic morphology.

#### Later processing: Lexeme processing

The anterior temporal lobe, specifically the superior temporal gyrus (STG) and middle temporal gyrus (MTG), has been implicated in the lexeme processing stage between 200–500 ms post-word onset in single word visual presentation.

For instance, Fruchter and Marantz (2015) found higher activity in the STG/MTG at 240–390 ms for words of stem derivational families with higher entropy, where stems with possible suffixes and a more equal spread of frequency among those have higher entropy. This effect, importantly, preceded whole-word frequency effects.

Besides derivational family entropy is semantic coherence, the discrepancy between the predicted and actual frequency of an existing word given its morpheme combinations (Fruchter & Marantz, 2015). Predicted frequency is calculated with variables which include the correlation of stems that combine with that affix and their respective frequencies, and a word’s phonological well-formedness estimated through bigram transition probabilities from stem to suffix. The difference (residual error) between the predicted and real frequency of a word is attributed to semantic coherence: the worse the semantic fit between stem and affix, the less the word would be used. Fruchter and Marantz (2015) found that words with lower semantic coherence correlated with increased orbitofrontal activity in the 380–500 ms time range.

The syntactic and semantic processing of the same morpheme has been differentiated as separate processes with different spatiotemporal signatures, with syntactic combination preceding semantic processing. Using a violation paradigm manipulating word-level syntactic versus semantic ill-formedness, Neophytou et al. (2018) found earlier effects of syntactic violations. Whereas syntactic effects occurred at 200–300 ms in the ventral and posterior temporal lobe (consistent with findings of King et al., 2016, on noun/verb, or NV, entropy), semantic effects occurred later between 300 and 500 ms in the orbitofrontal cortex (OFC; consistent with the finding of later activity for semantic world-knowledge word-level violations (e.g., “unflush”) after 400 ms in the ventromedial prefrontal cortex (Pylkkänen et al., 2009), as well as effects of semantic coherence in the OFC (Fruchter & Marantz, 2015).

### The Current Study

The importance of affix meanings for Arabic words allows for the prediction that affix class and its typical information content plays a role in lexical access. Building on previous findings about the processing of morphologically complex words using MEG, and given the more substantial information content of affixes in Arabic as compared to those of previously studied languages, we predict activity in areas implicated in lexical processing—namely, the inferior temporal gyrus (ITG), MTG, and STG, during lexical access and (re)combination of root and affix to be modulated by the relation of a word’s meaning to the typical meaning of its affix class.

For this study, following the functional definitions adopted in current Arabic psycholinguistic research, we use five affixes in Arabic. The first two affixes are nominal affixes that target semantic typicality, where the first affix (maCCaC) typically has a meaning of place and atypically has a meaning of tool, and the second affix (maCCaCa) typically has a meaning of tool and atypically has a meaning of place. This manipulation allows us to vary whether the combination of root and affix displays a typical or atypical meaning for the affix, while keeping the two meanings in question constant across the affixes.

The other three affixes are ones that manipulate syntactic category typicality, where the first of these unambiguously denotes a nominal form (C_1_aC_2_C_2_a:C_3_) (an agent), whereas the other two affixes are NV ambiguous and denote either nominals or active participles (Ca:CeC, muC_1_aC_2_C_2_eC_3_), with varying NV entropy. In parallel, we use two types of pseudowords across the five described affixes. The first class of pseudowords combines an existing root and affix, to create a word with no full word frequency (e.g., the equivalent of a word such as “cloudery”) but one that is generally consistent with the grammar of Arabic. The roots chosen for these words have a low productivity (in that they occur with a limited number of affixes). Importantly, in Arabic, since roots provide no grammatical information none of the pseudowords made from these roots violate grammatical category or argument structure, which was a distinction made and investigated in Neophytou et al. (2018). Given the frequency of these roots in other words and the frequency of the affixes, but the zero frequency of their combination, this class of words can be considered low on semantic coherence as defined by Fruchter and Marantz (2015; see section 2.4.2). Crucially, since these words are parsible into root and affix, some meaning could be coerced into them, and they are therefore *potential words*, although vague. The second class of pseudowords is more straightforward, as its words combine a phonotactically plausible nonexistent triconsonantal root and a target affix (e.g., the equivalent of a word such as “lopper”).

We employ a continuous visual lexical decision task presenting stimuli at a random order while recording neural responses using high-temporal resolution MEG. We opted for a visual paradigm to be in line with the customary modality for studies most related to the current one, as well as because Levantine Arabic varies in its pronunciation considerably across the region it is spoken in, and therefore among our participants. Investigating both semantic and syntactic variables in relation to affix productivity, we predict that words are decomposed into root and affix, with a semantic role for both, and centrally, that affix meaning typicality (more reliably mapping to one meaning) plays a facilitatory role in the processing of words during the recomposition stage of recognizing morphologically complex words. Specifically, for the words in the semantic typicality manipulation, we predict finding an early morphological decomposition effect between 50 and 200 ms at the left FFG, with an M170 response modulated by the frequency (or zero-frequency pseudoroots) of roots and affixes.

At the semantic composition stage, given the association of the STG/MTG with lexeme processing, we predict that the lexical access of root and affix to involve meaning typicality (a lexical feature of morphemes). Specifically, we predict an effect between 200 and 500 ms at the STG/MTG; with a higher processing cost for less typical words of an affix reflected in higher activity in this spatiotemporal area.

As for the syntactic typicality manipulation words in the early decomposition stage, we predict an early morphological decomposition effect between 50 and 200 ms at the left FFG similar to that of the semantic typicality manipulation words. At the composition stage, we predict a syntactic licensing effect at around 200 ms at the STG/MTG, with a higher processing cost for higher NV entropy words reflected as higher activity in this spatiotemporal area.

Concerning pseudowords, we predict an early morphological decomposition effect between 50 and 200 ms at the left FFG, with a higher activity in this spatiotemporal area for existing-root potential-word pseudowords as compared to nonroot pseudowords. At the semantic composition stage, we predict finding effects from 200 to 500 ms at the STG/MTG; with a higher processing cost for potential-word pseudowords compared to existing words, reflected as higher activity in this spatiotemporal area. Later, from 300 to 500 ms, in the OFC, we expect higher activity for potential-word pseudowords given their low semantic coherence.

## MATERIALS AND METHODS

### Participants

#### Behavioral tasks

Fourteen native speakers of Levantine Arabic who had spent a significant portion of their lives—including their childhood—in a Levantine-Arabic context participated in each of the two online tasks. Heritage speakers of the language were excluded, but diaspora speakers were included due to the current demographics of Levantine speakers.

The behavioral lexical decision task and semantic categorization tasks were hosted on the Gorilla Experiment Builder website (Anwyl-Irvine et al., 2020). After informed consent was obtained, demographic information concerning gender, age, and language background was collected.

#### MEG task

Participants in the MEG task were different from participants in the behavioral task, eliminating the chance of exposure bias. Thirty (9 female) native speakers of Levantine Arabic who have spent a significant portion of their lives—including their childhood—in a Levantine-Arabic context and are eligible to be recorded with MEG were invited to participate in this task. Four participants were omitted from analysis due to excessive movement, being a suspected heritage speaker, inattention and poor task performance during the experiment, and missing triggers. After that, 26 (8 female) participants were included in the analysis. Twenty-one participants spoke Lebanese Arabic, four spoke Palestinian Arabic, and one spoke Syrian Arabic. The average age was 31.4 years, ranging from 20 to 65 years (*SD* = 9 yr). Three participants were left-handed. All participants were Arabic-English bilinguals, partially due to the anglophone setting where they were recruited. After reading and understanding an informed consent form, the experimenter ensured that they understood the task and reminded them that this project is more interested in the Arabic language as it is used and understood daily, rather than how it is prescribed and corrected in grammar books. After the experiment, all participants were compensated $30 for their time spent participating in the experiment. Participants’ behavioral accuracy scores were checked before inclusion into analysis (>75% accuracy was required for inclusion). Participants were also given the option to receive their behavioral scores with an explanation, along with all the stimuli and their descriptions.

The New York University (NYU) Institutional Review Board approved both the online behavioral experiment and the MEG experiments, which we followed in accordance with the relevant guidelines and regulations.

### Stimuli and Design

#### Lexical decision task

After informed consent and demographic/linguistic information was collected, instructions were given for completing the lexical decision task. Participants were told to answer if a word is a real word or not as fast and as accurately as possible for every word presented on the screen. Behavioral experiment participants were instructed to click 1 if it was a word, and 0 if it was not a word. MEG participants were given a four-button joystick to record their responses and instructed to use either of the first two buttons: leftmost (blue) button if it was not a word and the second button (yellow) if it was a word. Participants were told to use their initial judgment in deciding if something is a real word or not and were told that deciding if a word is real does not necessitate that they know what it means, but rather that it could be meaningful and used in some context. Each participant was presented with 660 total stimuli in a lexical decision task.

**Figure 1.**
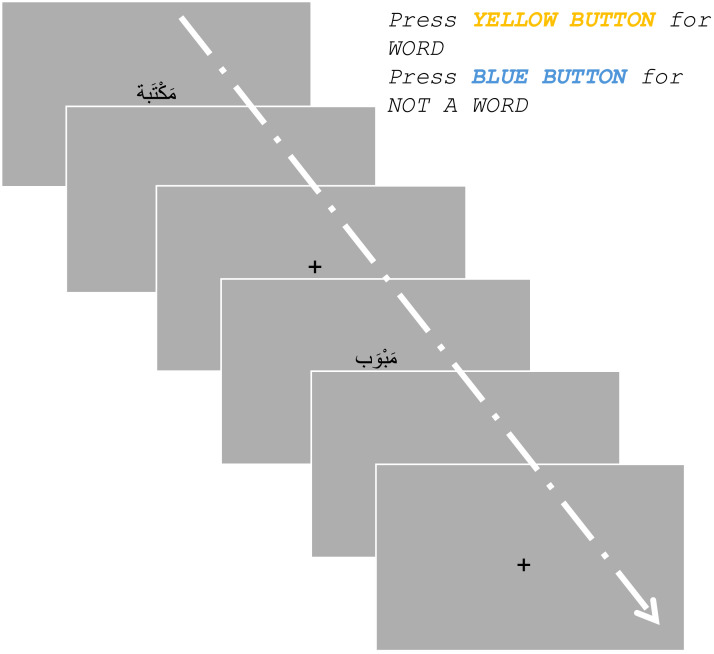
Lexical decision task sequence. Every word (of any category from designs 1 and 2) is preceded by a blank screen, then a fixation cross. For every word, the participant has an indefinite time window to decide if it is a word or not by pressing on one of two buttons. Then a blank screen followed by a fixation cross appears before the next stimulus. This sequence is repeated until all stimuli have been shown to the participant (165 for behavioral participants and 660 for MEG participants). The presentation is similar for both the behavioral and MEG tasks. For the MEG participants, after every 55 words for a total of 12 blocks, there is a break opportunity (of indefinite time), which the participant may choose to take or not. Participants were encouraged to take the break to rest their eyes and mind off the task but reminded not to move.

For the behavioral task participants, each was shown 165 out of the 660 stimuli for higher chances of participant recruitment and task engagement. For every word, a blank screen was presented for 300 ms, then a fixation cross for 250 ms followed by the target word centered on the position of the cross, and the target item remained present until the participant made their choice (see Figure 1). For the MEG participants, the task lasted between 35 and 50 minutes. For the behavioral task participants, it lasted between 7 and 9.5 minutes. The manipulation in this task is that of the functional morpheme of the words and their (1) semantic or (2) syntactic typicality.

Although later analyzed separately, the two groups of stimuli from experiment design 1 and experiment design 2 (explained below) are interleaved within the same blocks to increase the validity and reliability of the individual stimuli. Otherwise, there would be a possibility of desensitization due to presented words all having the same two (in experiment 1) or three (in experiment 2) functional morphemes, which leads to a great deal of visual uniformity among the stimuli. For the sake of clarity, each experiment’s design rationale will be explained separately.

##### Experiment design 1: Semantic typicality of nominal affix morphemes.

Two dissociative patterns were chosen to contrast against each other in semantic category typicality. These two functional patterns are maCCaCa (form 1.1) and maCCaC (form 1.2). The letter C represents a root consonant, and the functional morpheme is the vocalic pattern and specified consonants (ma__a_; ma__a_a). Both patterns 1.1 and 1.2 are nominal and are not syntactically ambiguous when presented with diacritics.

The critical manipulation relies on the following: most words within the family of pattern 1.1 denote tools, such as “*mamsaħa*” (“mop” [root MSĦ denotes “wiping”]), “*mabhara*” (“peppermill” [BHR: “pepper”]), “*manshafe*” (“towel” [NShF: “drying”]). A smaller family of words within pattern 1.1 denote places such as “*madrase*” (“school” [DRS: “study”]), “*masmake*” (“fish market” [SMK: “fish”]), “*mazbale*” (“dumping ground” [ZBL: “trash”]). The reverse is true for words with pattern 1.2, which typically denote a place, such as “*maktab*” (“office” [KTB: “write”]), “*maʕmal*” (“factory” [ʕML: “work”]), “*malja*” (“shelter/refuge place” [LJA: “refuge”]). Words with pattern 1.2 atypically denote tools, such as “*manħat*” (“chisel” [NĦT: “sculpt”]), “*malqat*” (“tweezers” [LQT: [“catch”]), “*mabrad*” (“nail file” [BRD: “file” (v)]). Note that there are other semantic categories derived with these patterns, such as abstract concepts (e.g., “*masʔala*” [“issue”] for pattern 1.1 and “*marjaʕ*” [“reference”] or “*mabdaʔ*” [“concept”] for form 1.2). Nevertheless, the dissociative semantic categories of tool and place we have chosen are still the norm for each of the patterns 1.1 and 1.2, respectively. To further consolidate and quantify this, a norming task for the semantic categorization of these stimuli, including pseudowords with these patterns, was conducted (see Semantic Category Quantification: Behavioral Semantic Categorization Task).

As for the pseudoword condition, all words also follow patterns 1.1 and 1.2 and are divided into two categories. The first category contains novel combinations of existing roots with these patterns. The meaning of these novel words is not immediately available to speakers. The roots of these words exist but not in this pattern; that is, the words have zero frequency, which was checked across online dictionaries, as well as with a varied sample of Levantine Arabic speakers who were consulted for these stimuli (varied in age and area of the Levant). This latter step is necessary, since Arabic corpora are incomplete and might not contain words and roots that are present in spoken variants of Arabic. Examples include “*manhara*” (NHR: “river”; pattern 1.1). In this example, if one were to coerce meaning out of this word, it is not clear whether this is something that makes rivers or a place where rivers are or where rivers are made. For pattern 1.2, an example is “*magyam*” (GYM: “cloud, fog”; pattern 1.2). In this example as well, it is unclear what this word might denote. In general, these words are parsible but not semantically transparent.

The second category of pseudowords is more straightforward. The roots do not exist but follow phonotactic rules of Levantine Arabic and therefore are pronounceable. Without an existing use, semantic information is absent from the root. The only early semantic information that could be extracted from such words is from the affix patterns 1.1 or 1.2. Similarly, as in the previous pseudoword condition, to ensure their nonexistence, after their design following the phonotactic restrictions of Arabic and judged plausible, nonroots were searched in corpora and online dictionaries in the past simple form (CaCaCa, a highly common occurrence of roots in words) as well as with native speakers, given the prevalence of colloquial words absent in corpora. With atypical words being a smaller category of the affix to start, the number of potential stimuli is smaller. The average frequencies across conditions are matched, though the number of stimuli per condition could not be matched across conditions. This was done in the aim of creating a rich dataset for the possibility of later analyses, different from the present analysis. The design is laid out in Table 5.

**Table 5.**
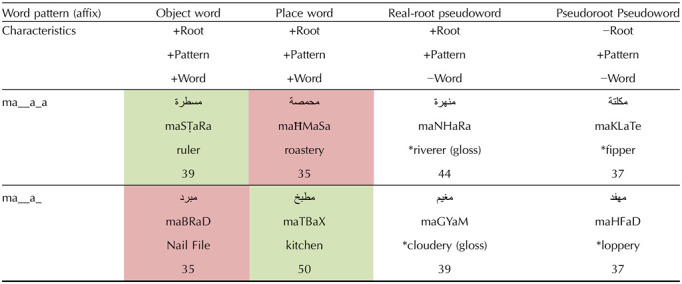
Semantic typicality manipulation design (numbers indicate stimuli per category)

*Note*. 1. Cells in green: typical meaning-to-affix relation. Cells in red: atypical meaning-to-affix relation. 2. A “*” preceding a word indicates a pseudoword.

##### Experiment design 2: Syntactic noun/verb typicality and functional morpheme ambiguity.

Three distinct word patterns were chosen to contrast with each other in syntactic ambiguity and nonambiguity, where two were NV ambiguous, and one was uniformly nominal. The ambiguous patterns were Ca:CeC (pattern 2.1.1) and muCaCCeC (pattern 2.1.2). Both are at times nominal forms, and at other times participles. They were contrasted to the unambiguous nominal pattern CaCCa:C (pattern 2.2). Note that sometimes, this pattern is a plural inflection of the nominal pattern Ca:CeC. Nevertheless, in both cases, this pattern would be nominal. To complement experiment 1, all three patterns were kept constant for the semantic category of agent when in nominal form. The stimuli chosen were ones where the semantic shift is minimal between the verbal and nominal forms (e.g., for pattern 2.1.1, Ka:TeB: nominal: writer or active participle: writing/written; for pattern 2.1.2, mNaSSeʔ nominal: organizer; causative active participle: organizing/organized). Therefore, the difference among the patterns is largely regarding syntactic ambiguity and less about semantic ambiguity. In contrast, an unambiguous nominal pattern, CaCCa:C (form 2.2) also denotes an agent but cannot be a verb. Importantly, pattern 2.2 is found in several Levantine Arabic words which have different nominalizing patterns in modern standard Arabic. For a homogeneous sample and to preserve the same perceived frequency and legitimacy of our stimuli, we restrict our studied population to speakers of Levantine Arabic. A more restricted sample will allow for more secure generalizations, especially since morpheme productivity is heavily dependent on language variants. Arabic variants have different grammars, as well as distributions and productivities of grammatical rules.

As for the pseudoword conditions that match the syntactic ambiguity manipulation, they follow the same logic as for the pseudowords of experiment 1. That is, one set uses existing roots with the 2.1.1, 2.1.2, and 2.2 patterns to produce non-existing words, while the second set uses phonotactically plausible nonroots with these patterns. There are a minimum of 40 stimuli per form class (43 for 2.1.1, 45 for 2.1.1, and 61 for 2.2), with combined pseudowords matched in count for each form class. The stimuli were collected by crowdsourcing and correspondence with other native speakers of Levantine Arabic, and as many words were collected for inclusion, with the expectation that some may have to be removed after frequency intuition data was collected. For this reason, the number of words is not equal across conditions. The design is laid out in Table 6.

**Table 6.**
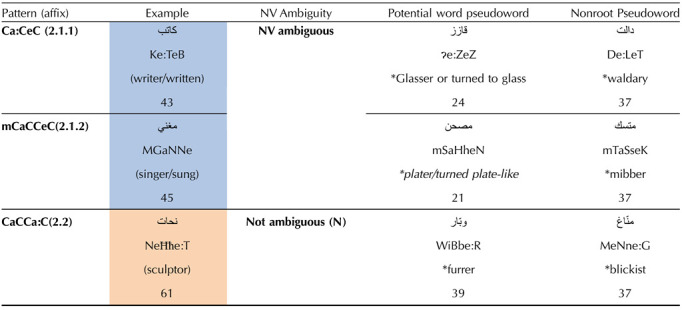
Syntactic manipulation design (numbers indicate stimuli per condition)

*Note*. 1. English translations of last pseudoword items are approximations and do not demonstrate noun/verb (NV) ambiguity. 2. A “*” preceding a word indicates a pseudoword.

##### Semantic category quantification: Behavioral semantic categorization task.

For experiment 1, semantic category is a crucial measurement in our manipulations. However, unlike frequency and syntactic category, semantic category is not a variable that is commonly annotated in corpora. To add, semantic categories are not clearly delineated: a word may fit several semantic categories, where it may be a better fit for one category (e.g., tools: “hammer”; places: “office”) and a worse fit for another (e.g., tools: “stone”; places: “desk,” which need to be contextualized for a better fit, e.g., “I left your glasses *on the desk*”; “He sharpens his knives with this *stone*”). Within our stimuli, different items may have different semantic fits to the categories we are contrasting, place and tool. Consequently, to quantify semantic categories, we used an online semantic categorization crowdsourcing task.

Twenty-four participants were randomized into place categorizers or tool categorizers and asked to rate each word as a member of their designated category on a 1–7 Likert scale. To increase the ecological validity of the task, each category was rated separately and by different raters, with the participant blind to the alternative rating category. This would prevent participants from assessing categorization as a dichotomy between place and tool, which are not categories that are inherently mutually exclusive. We ruled out alternative measures such as forced choice between place or tool, and a measure where each category is an extreme of the same scale, since the middle could represent either *neither place nor tool* or *both place and tool*. Additionally, participants were also asked to categorize the existing-root nonwords described previously in the experiment 1 design section. Through this task, we were able to capture participants’ overt intuitions of the semantics of our chosen functional morphemes 1.1 and 1.2, since they have no previous semantic information about these potential-word pseudowords other than the vague, polysemous meanings of the root and the pattern.

Each item’s score was averaged across participants to obtain its individual categorization score for the place and the tool categories. This continuous semantic categorization score is a more sensitive measure than binning into discrete categories and is used for the analysis of behavioral measures on the task.

#### Frequency quantification and Levantine corpus data

The paucity of corpora for Arabic, particularly Levantine Arabic, limits the ability to obtain frequency tags for all words. Instead, frequency intuitions were collected for our items on a 1–7 Likert scale for all words of experiments 1 and 2. Word familiarity has been found to correlate more highly with spoken word corpora than written word corpora (Tanaka-Ishii & Terada, 2011). This distinction is especially pertinent to our studied variant of Arabic, since Levantine Arabic is a largely spoken variant, and is less commonly written, especially in formal settings. Moreover, frequency or familiarity judgments (a participant’s judgment of frequency of exposure to a word; Stadthagen-Gonzalez & Davis, 2006) have been interpreted as equivalent to the corpus frequency of a word (MRC Psycholinguistics Database, 2006), to which they were shown to be highly correlated (Li, 2013). In total, 12 participants (native speakers of Levantine Arabic) completed the frequency intuition survey. Metrics of frequency are presented in Table 7, with significant differences as a pairwise *t* test indicated across items.

**Table 7.**
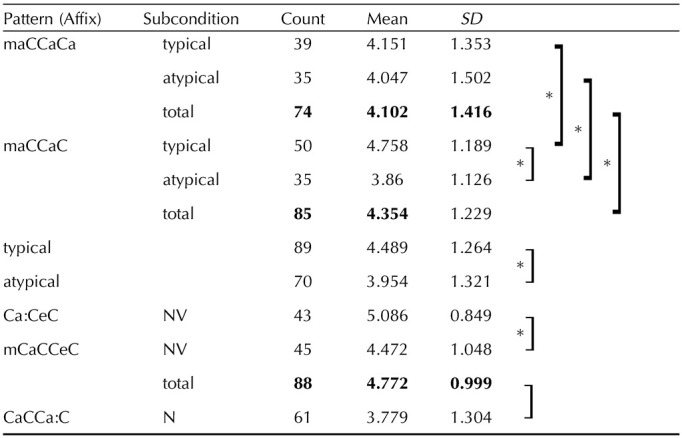
Summary of mean frequency intuitions

*Note*. **Boldface** indicates totals.

### Procedure

Before the MEG experiment, the participant’s head shape was digitized using a handheld FastSCAN laser scanner (Polhemus, VT, USA). The digitized head shape was later used in the data preprocessing stage for co-registration of the MEG sensors with the participant’s head. Five points on the participant’s head were marked with a marker then digitized: three on the forehead (center, left, and right) and two anterior of each ear’s auditory canal. Before entering the magnetically shielded room (MSR), an explanation about the task was given to the participant, after which they completed a short practice session with feedback on the accuracy of their answers. The experimenter was present as they ran the practice session to answer any questions. After we made sure that the task had been understood, participants were guided inside the MSR, where marker coils were placed on the previously digitized marker points, in order to localize each participant’s head with respect to the MEG sensors. Marker location measurements were obtained right before and right after the experiment, thus providing a measure of overall movement during the recording session. Participants were given a total of 11 break opportunities (every 3–5 min, after the completion of a block) and were told that they could choose to take them or move on to the next block. There was a total of 12 blocks, with 55 randomized unique words per block, for a total of 660 randomized unique words.

We used a projector that relayed the image onto a screen inside the MSR at approximately 0.7° vertically. We used PsychoPy3 (Peirce et al., 2019), the Python Arabic Text Reshaper package (https://github.com/mpcabd/python-arabic-reshaper) for the presentation of the stimuli, as well as the Sahel Regular font for the display of Arabic diacritics (which were necessary for our experiment; https://arabicfonts.net/fonts/sahel-regular). Content was presented in black against a gray background. Each trial began with a fixation cross that appeared in the center for 250 ms. Each word appeared in the center and stayed onscreen until the participant pressed either button (word/not a word), followed by a blank screen for 300 ms, before the onset of the next fixation cross and word. Participants were instructed to focus during the experiment, to avoid excessive blinking or moving as much as possible and were encouraged to use the frequent breaks for blinking, resting eyes, and minor adjustments not involving the head.

During the experiment, MEG data were acquired continuously using a 157-channel axial gradiometer system at the KIT/NYU MEG lab (Kanazawa Institute of Technology, Kanazawa, Japan), with a sampling rate of 1000 Hz and while applying an online high-pass filter of 0.1 Hz and low-pass filter of 200 Hz. The experimental runs lasted approximately 35–50 minutes, and participants were paid for their participation.

#### MEG data preprocessing

MEG data underwent preprocessing prior to being used for analysis. First, the data were noise-reduced using the continuously adjusted least square method (Adachi et al., 2001) provided in the MEG160 software (Yokohawa, Electric Corporation and Eagle Technology Corporation, Tokyo, Japan); this discounts noise recorded by three magnetometer reference channels located away from the participant’s head from the data of interest. The noise-reduced data were imported into MNE-Python (Gramfort et al., 2013; Version 1.7.0) and Eelbrain (Brodbeck et al., 2023; Version 0.25.1), where they were band-pass-filtered between 1 Hz and 40 Hz. Following standard lab procedures, data from bad channels (flat or excessively noisy channels; number in NY: 2–3) were overridden and interpolated from the remaining sensors using MNE’s implementation of the spherical spline interpolation method (Perrin et al., 1989). After interpolating the bad channels, an independent component analysis (ICA) algorithm was then applied to the data. The ICA results helped identify and remove noise-related components based on visual inspection of the spatial and temporal profiles of these components. We only removed noise components that were identifiable (eye blinks, heartbeats) or characteristic of the MEG system in the laboratory. The data were then segmented into epochs, from 200 ms before the onset of the word until 600 ms post-onset. Baseline correction was applied to each epoch based on the 200 ms of data that preceded each trial. Epochs containing signal amplitudes that exceeded a threshold of ±3,000 femotesla were automatically rejected from the analysis.

When participants did not have an MRI scan collected at the NYU Center for Brain Imaging (NYU CBI), we scaled the FreeSurfer average brain (“fsaverage” brain; available in the FreeSurfer software suite: https://surfer.nmr.mgh.harvard.edu; Fischl, 2012) to match each participant’s digitized head shape. From this we created a source-space consisting of 2,562 vertices per hemisphere. Using the boundary element model method, the activity at each vertex was used to calculate the forward solution. The inverse solution was then estimated for each subject, using a signal-to-noise ratio value of 3. With the majority of our participants having been co-registered to the FreeSurfer average brain instead of an anatomically accurate MRI scan, we opted to report results for the unsigned, free orientation source reconstruction scheme, which imposes no constraints on dipoles’ orientation with relation to the cortical surface, although we ran our analyses on signed data as well. Activation estimates were obtained from the magnitude of each dipole, ignoring its orientation. The inverse solution resulted in a noise-normalized dynamic statistical parameter map (Dale et al., 2000). We defined the VWFA associated with form-based processing as Brodmann Area (BA) 37, the temporal pole associated with form and meaning processing as BA 38, and defined the spatial window associated with meaning-based processing as BAs 20, 21, and 22. Together, these defined our temporal cortex search window. BAs 44 and 45 were defined as areas associated with semantic and world-knowledge based processing.

#### Statistical analyses

We analyzed the MEG data using a spatiotemporal cluster-based permutation test, identifying clusters of contiguous vertices and timepoints for which the statistics derived from the estimated activation levels were jointly significant. The test was conducted on the full spatiotemporal activation time-course within each region of interest (ROI). The cutoff for a time point contributing to a cluster was a *p* value of 0.05.

For all statistical tests reported, we conducted a subsequent cluster-based permutation test (following Maris & Oostenveld, 2007), comparing any resulting clusters against a distribution generated from the null hypothesis, based on 10,000 random permutations. For each permutation, each participant’s condition labels were independently and randomly permuted.

## RESULTS

### Behavioral Results

Before running any analyses, we omitted participants with a performance score below our cutoff of 75% accuracy on real words. Two people were rejected based on this criterion. We cleaned all data, first removing trials with reaction times (RTs) deemed unreasonable (under 200 ms and over 5 s long), and then removing any outlier data falling 3 *SD*s beyond the mean (within-participant) for each lexicality type. Additionally, we compared the results between online participants and MEG task participants. A *t* test revealed a significant difference in the RTs between the online group (*M* = 1.6 s) and the MEG group (*M* = 1.52 s) (*t*(114,477) = 5.75, *p* < 0.001). Additionally, a *t* test revealed a significant difference between the accuracies of each group: This was true generally and across lexicality conditions. The results follow, indicating online group and MEG group means, respectively: General: *M* = 78%; 82% (*t*(10,257) = −6.2149, *p* < 0.001); words: *M* = 88%; 82% (*t*(3,759.6) = 7.104, *p* < 0.001); existing-root pseudowords: *M* = 58%; 68% (*t*[3,759.7] = −7.67, *p* < 0.001); and nonroot pseudowords: *M* = 89%; 94% (*t*[3,040] = −7.26, *p* < 0.001).

#### Behavioral data of MEG task participants

**Figure 2.**
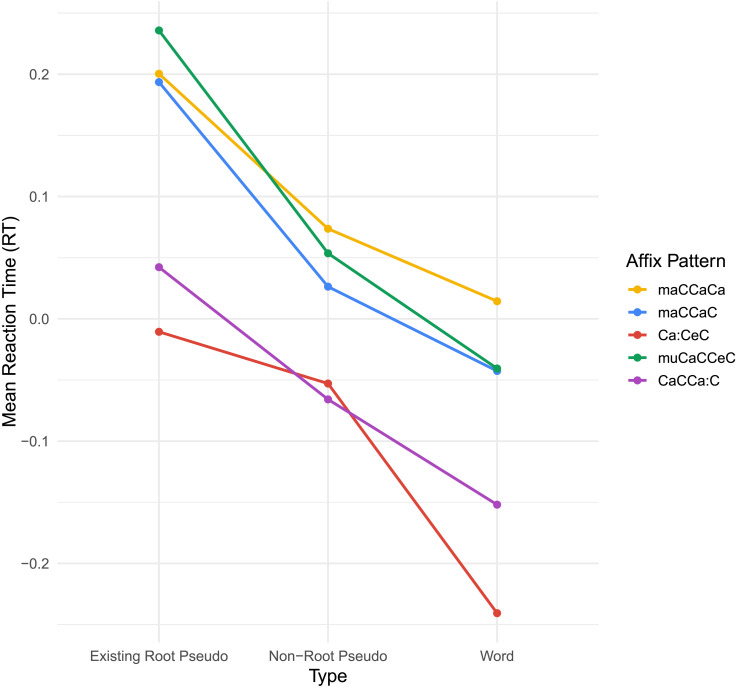
Comparison of reaction times (RTs) across affixes. C and E are the shortest in length and have the fastest RTs.

The cleaned descriptive data for RTs and accuracy measures for the different categories of words are presented in Table 8. As predicted, the existing-root pseudowords had the longest RTs and lowest accuracy scores for all classes of words. Additionally, there is an effect of affixes, not sufficiently explainable by word length and possibly explainable by the position of root letters with respect to the affix letters. As described in the Introduction, words of one affix all have the same word length, so all our stimuli had four (affixes B, C, D, E) or five letters (affix A). Affixes (patterns) C and E had faster RTs (1.25 s and 1.36 s, respectively, relative to mean = 1.45 s), than other affixes of similar length (4 letters; affixes B and D), as illustrated in Figure 2. There is a main effect of lexicality, as the same trend is observed across affixes.

**Table 8.** Descriptive means of relative accuracy and reaction time divided by affixes, lexicality, and syntactic ambiguity

Affixes		Relative accuracy				Reaction time (s)			
		Lexicality							
		Words	NW	NR	Total	Words	NW	NR	Total
A. maCCaCa	typical (A1)	75%	57%	93%	75%	1.631	1.857	1.653	1.664
	atypical (A2)	79%				1.474			
	total	77%				1.557			
B. maCCaC	typical (B2)	81%	67%	94%	80%	1.425	1.791	1.592	1.581
	atypical (B1)	76%				1.565			
	total	79%				1.483			
C. Ca:CeC		94%	83%	94%	91%	1.193	1.488	1.32	1.306
D. mCaCCeC		91%	65%	94%	87%	1.497	1.871	1.639	1.623
	total	92%	75%	94%	89%	1.347	1.666	1.479	1.463
E. CaCCa:C		79%	72%	96%	82%	1.319	1.605	1.393	1.42
Total		82%	68%	94%	82%	1.429	1.731	1.519	1.529

*Note*. NW = nonword; NR = nonroot.

#### Semantic categorization task

**Figure 3.**
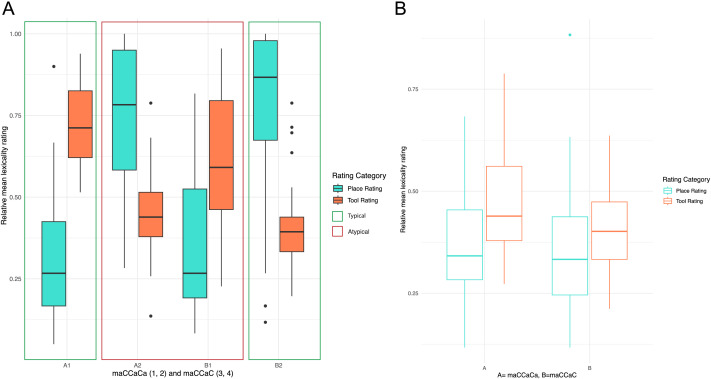
Comparisons of semantic category fit. (A) Fit for real words, divided by affix and typical and typical categories. (B) For pseudowords divided by affix and typical and atypical categories (see Table 8).

The results of the semantic categorization task are presented in Table 9 and illustrated in Figure 3. Typical words (A1 and B2) show a larger difference in their ratings as places versus tools, while atypical words (A2 and B1) have closer ratings between the two categories. For pseudowords, there was no significant difference in category ratings across affixes. From the behavioral data, there appears to be no inherent bias in understanding each affix as indicating place or tool. However, there is a general bias toward categorizing words as tools rather than places. This bias would need further investigation to see if the tool category is a more open category than the place category. Another alternative is that the categorization is more heavily influenced by the meaning of roots themselves, which by design had low productivity and therefore less variation in meaning across words in which those roots occur.

**Table 9.** Semantic categorization descriptive results for words and nonwords

	**Place fit**		**Tool fit**	
	Mean	*SD*	Mean	*SD*
maCCaCa typical	0.297	0.163	0.73	0.125
maCCaCa atypical	0.753	0.209	0.453	0.134
**maCCaCa**	0.516	0.294	0.6	0.19
maCCaC typical	0.786	0.237	0.396	0.116
maCCaC atypical	0.353	0.22	0.607	0.215
**maCCaC**	0.613	0.311	0.484	0.191
**Typical**	0.581	0.316	0.542	0.203
**Atypical**	0.551	0.294	0.533	0.195
**Tool words**	0.333	0.204	0.669	0.181
**Place words**	0.771	0.223	0.424	0.131
maCCaCa nonword	0.37	0.137	0.473	0.126
maCCaC nonword	0.356	0.15	0.401	0.093

*Note*. **Boldface** indicates totals.

#### Lexicality

We ran a linear regression predicting RT based on lexicality interacting with affix identity as well as the frequency of existing words. Because, in Arabic, words sharing an affix have the same length and root position, we excluded them from our regression models. These are inevitably conflated with affix identity and therefore vary only in 1 of 5 of the affixes. Nevertheless, we attempted to include them in our analyses, but the model failed to converge due to the collinearity of each of these with affix identity. The model is described with the following formula:

ReactionTime=Intercept+Lexicality+Affix+Lexicality*Affix+Frequency (1)

The model was significant, *F*(15, 19318) = 89.58, *p* < 0.0001, at *R*^2^ = 0.065 (adjusted = 0.064), with residual standard error = 0.4715. Lexicality showed an overall main effect, with a larger effect of nonroot pseudowords than real-root pseudowords. Affixes C and E showed a significant speeding effect on RT, as did frequency. As for interaction effects, affix C significantly interacted with lexicality type, showing faster RTs for both pseudoword conditions. Next, we ran a logistic regression for accuracy with the same variables, described as:

$$\text{Accuracy}=\text{Intercept}+\text{Lexicality}+\text{Affix}+\text{Lexicality}*\text{Affix}+\text{Frequency}+\left(\text{Intercept}|\text{Participant}\right)$$ (2)

After the model failed to converge twice, we first removed affix as a main dependent variable, since it reoccurs as an interaction variable, and subsequently removed frequency intuition, since it is peripheral to the aim of this lexicality model, especially given the other variables. The model fit was evaluated using the Akaike information criterion (AIC: 16,103.7) and Bayesian information criterion (BIC: 16,229.6). The log-likelihood of the model was −8,035.8, and the deviance was 16,071.7, with 19,318 residual degrees of freedom. Similarly to the RT model, affix C showed significant positive effects on accuracy. Nonroot pseudowords also had a larger effect on accuracy than real root pseudowords. The full results of both models are summarized in Supplementary Table S11.

#### Semantic typicality

**Figure 4.**
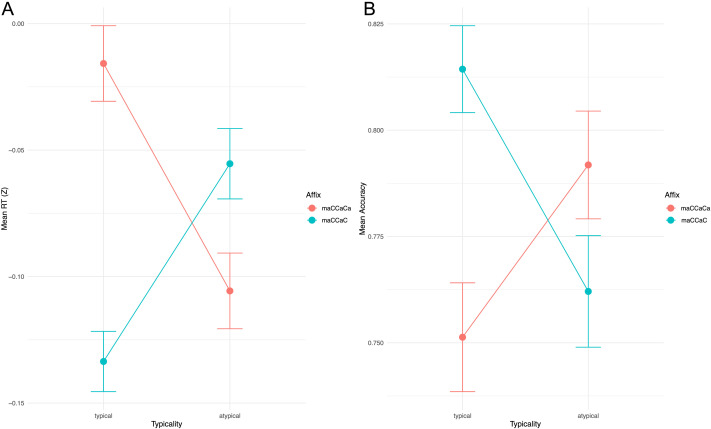
Comparisons of reaction time and accuracy between typical and atypical scores. (A) Reaction time. (B) Accuracy.

As elaborated in the Introduction, for each of our two contrasted affixes A and B, there is one typical semantic category, which is the atypical category for the other. Descriptive means for these typicality responses are illustrated in Figure 4. According to our hypothesis, we would expect consistent lower RT and higher accuracy for typical words, which would show as parallel red and blue lines. However, though very small for RT in particular, we found an interaction between affix and typicality.

We conducted a linear regression model for existing words of affixes A and B to examine whether semantic typicality (categorically: typical vs. atypical) predicts RT and another predicting accuracy. For both models, the dependent variables were typicality-atypicality interacting with affix type, alongside frequency:

$$\text{Reaction}\;\text{Time}=\text{Intercept}+\text{Typicality}+\text{Affix}+\text{Typicality}*\text{Affix}+\text{Frequency}+\text{Typicality}*\text{Frequency}+\left(\text{Intercept}|\text{Participant}\right)$$ (3)

$$\text{Accuracy}=\text{Intercept}+\text{Typicality}+\text{Affix}+\text{Typicality}*\text{Affix}+\text{Frequency}+\text{Typicality}*\text{Frequency}+\left(\text{Intercept}|\text{Participant}\right)$$ (4)

The model was significant, *F*(5, 4668) = 75.3, *p* < 0.0001, at *R*^2^ = 0.0746 (adjusted = 0.0737), with residual standard error = 0.7969. Expectedly, higher frequency led to lower RTs, but there was no main effect of typicality, and there was a significant main effect of affix B. The interaction between typicality and affix type was significant, with an interaction effect between typicality and affix B predicting faster RT. The accuracy logistic regression model was evaluated using AIC (3,789.5) and BIC (3,834.7). The log-likelihood of the model was −1,887.8, and the deviance was 3,775.5, with 4,667 residual degrees of freedom. Expectedly, since these are all existing words, the only significant effect was frequency, which positively predicted accuracy. The full results are summarized in Supplementary Table S12.

#### Exploratory analysis: Continuous semantic category fit

Finally, we conducted an exploratory analysis using our semantic categorization task fits and ran similar regression models to the above, with typicality defined as a continuous variable of semantic fit for each place category and tool category, as described in Materials and Methods. The two models are defined as the following:

ReactionTime=Intercept+Placefit+Toolfit+Affix+Placefit*Affix+Toolfit*Affix+Frequency (5)

$$\text{Accuracy}=\text{Intercept}+\text{Place}\;\text{fit}+\text{Tool}\;\text{fit}+\text{Affix}+\text{Place}\;\text{fit}*\text{Affix}+\text{Tool}\;\text{fit}*\text{Affix}+\text{Frequency}+\left(\text{Intercept}|\text{Participant}\right)$$ (6)

**Figure 5.**
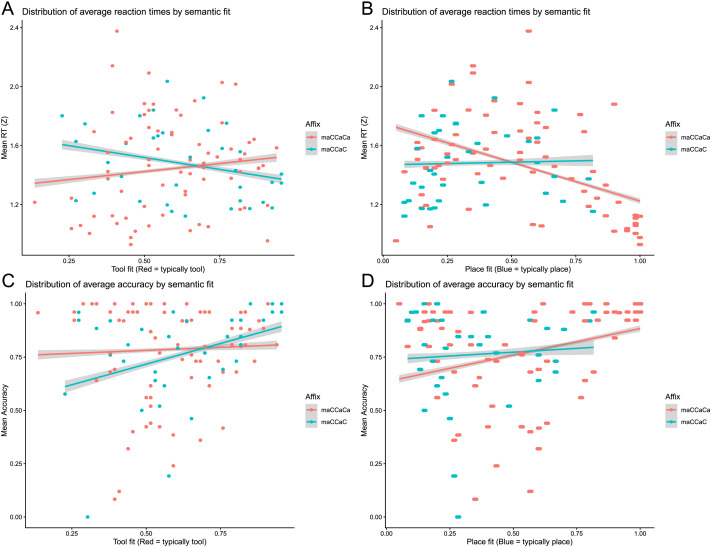
Distribution of averages for reaction time (RT) and accuracy by semantic fit. (A) RT regression fit for Place fit. (B) RT regression fit for Tool fit. (C) Accuracy regression fit for Place fit. (D). Accuracy regression fit for Tool fit.

The RT model was significant, *F*(6, 4649) = 68.49, *p* < .0001, at *R*^2^ = 0.0821 (adjusted = 0.080), with residual standard error = 0.7941, with a similar but improved fit compared to the previous model with typicality as two categorical variables. Alongside frequency, better place fit significantly predicted faster RT, unlike tool fit which was not significant. As for the accuracy model, AIC was = 3,689.5, BIC = 3,741.1. The log-likelihood was −1,836.8, with a deviance of 3,673.5 and 4,648 residual degrees of freedom. Similarly to the RT model, frequency and place fit (but not tool fit) significantly predicted accuracy, although for this model, affix B also had a significant effect, similarly for an interaction effect between tool fit and affix B. The results are illustrated in Figure 5, and the full results are summarized in Supplementary Table S13.

#### Syntactic ambiguity

We conducted separate linear regressions to predict RT and accuracy based on NV ambiguity (ambiguous vs. unambiguous) interacting with affix type and frequency, described in the formula below:

ReactionTime=Intercept+SyntacticAmbiguity*Affix+Frequency (7)

$$\text{Accuracy}=\text{Intercept}+\text{Syntactic}\;\text{Ambiguity}*\text{Affix}+\text{Frequency}+\left(\text{Intercept}|\text{Participant}\right)$$ (8)

The RT model was significant, *F*(3, 4403) = 83.2, with *R*^2^ = 0.054, (adjusted = 0.053). As with other models, frequency significantly predicted faster RT, and affix D predicted a lower RT. No effect of ambiguity was found for RT. As for the accuracy model, it was assessed with AIC (2,673.9) and BIC (2,705.8), with a log-likelihood of −1,331.9, deviance of 2,663.9 and 4,402 residual degrees of freedom. As for the accuracy model, frequency was a significant positive predictor, with more frequent words having higher accuracy, but affix and syntactic ambiguity were not significant predictors. The full model results are summarized in Supplementary Table S14.

### MEG Results

We divided our spatiotemporal cluster analyses temporally into an early window at 100–250 ms, associated with form-based processing, and a later window, at 250–500 ms, associated with meaning-based processing. We searched across the temporal pole, which included the VWFA within the FFG (BA 37), the temporal pole (BA 38), and the area comprising the ITG, MTG, and STG (BAs 20, 21, and 22). In these areas, for syntactic ambiguity, we predicted higher activity for NV ambiguous words early in the FFG, associated with form-based processing, and later in the MTG, associated with syntactic licensing, and higher activity for typical words later in the MTG/STG, associated with semantic composition. Additionally, for semantic typicality, we included the analysis of the inferior frontal gyrus (IFG), defined as BAs 44 and 45 for world-knowledge and semantic processing, and the OFC (BA 47), associated with the processing of semantic well-formedness.

#### Affix semantic typicality

**Figure 6.**
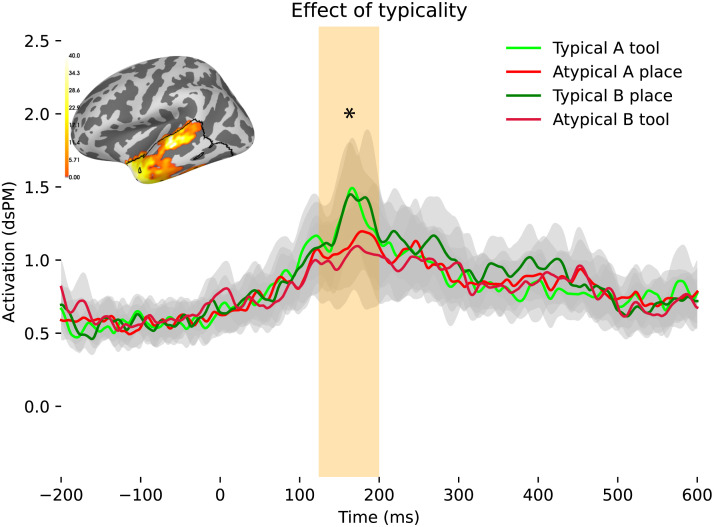
Main effect of typicality at the temporal pole, inferior temporal gyrus, and middle teimporal gyrus at 125–199 ms PSO, *p* = 0.0355. dSPM = dynamic statistical parameter map.

**Figure 7.**
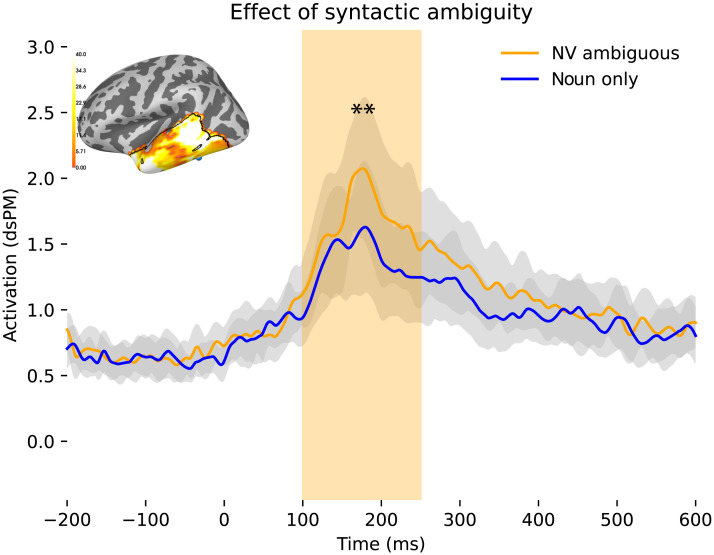
Higher activity for noun/verb ambiguous words across the temporal lobe all throughout the time window of interest, 100–250 ms, *p* = 0.0125. dSPM = dynamic statistical parameter map.

**Figure 8.**
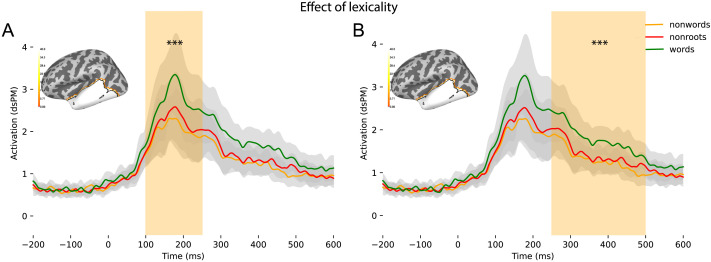
Effect of lexicality in the early and later windows of processing, with significantly higher activity for existing words than pseudowords. (A) 100–250 ms PSO (*p* < 0.0001). (B) 250–500 ms PSO (*p* < 0.0001). No significant difference was found between the activity of the two kinds of pseudowords in a temporal lobe-wide search. dSPM = dynamic statistical parameter map.

We performed a repeated measures analysis of variance (rmANOVA) spatiotemporal cluster test for our 2 × 2 design of Affix × Typicality for semantic typicality in our defined temporal cortex search area (demarcated in Figures 6–8) for each of our two time windows. We found a main effect for semantic typicality: There was a significant effect spanning the temporal pole, ITG, and MTG, which the spatiotemporal cluster test reported at 125–199 ms post-stimulus onset (PSO), *p* = 0.0355, with higher activity for typical words, as illustrated in Figure 6. No significant effects were observed for either affix or semantic category effect alone, and no significant effects were found in the later time window.

#### Semantic category

Additionally, we ran spatiotemporal permutation cluster tests comparing place words and tool words from our semantic typicality stimuli in our specified ROI across our two time windows. No significant effects were found. In an exploratory test, we found a significant effect at 360–410 ms PSO (*p* = 0.0493) in BAs 41 and 42. These areas have been previously included as part of the ROIs within the temporal lobe, comprising BAs 37, 38, 20, 21, 22, 41, and 42 (see Neophytou et al., 2018). However, this effect is not significant in a search that encompasses the whole of the temporal lobe and does not survive correction for multiple comparisons.

#### Syntactic ambiguity

We ran we ran spatiotemporal permutation cluster tests comparing a difference between NV ambiguous words and unambiguous nominal words in our specified ROI across our two time windows. We found a significant cluster at the early time window of 100–250 ms, spanning throughout the spatial and time windows, as the test reports at 100–250 ms PSO, *p* = 0.0125, with higher activity for NV ambiguous words than unambiguous noun words, as shown in Figure 7. The spatial and temporal extent of this finding suggests a sustained effect of syntactic processing early on in the temporal lobe. However, at our later time window of 250–500 ms, results were not significant. Given that BAs 41 and 42 have been included in the definition of the temporal lobe previously (Neophytou et al., 2018), we performed an exploratory spatiotemporal test for that area at *t* = 250–500 ms and found a significant effect of syntactic ambiguity between 250–278 ms PSO, *p* = 0.043. This last result, however, does not survive correction for multiple comparisons.

#### Lexicality

We ran a one-way ANOVA with three levels for words, real-root pseudowords, and nonroot pseudowords in our specified ROI across our two time windows. At the early time window of 100–250 ms, we found a sustained effect of lexicality across the whole time window throughout the temporal lobe (*p* = 0.0001), with a cluster being found all throughout the time window indicating more activity for existing words. For the later time window of 250–500 ms PSO, we obtained the same trend of significant results showing a sustained effect of lexicality associated with a significant cluster across the temporal lobe and all throughout the time window of 250–500 ms. These early and later sustained effects are illustrated in Figure 8. Additionally, we found a significant effect at 294–500 ms PSO in the IFG (*p* = 0.0001), with more activity for words.

We then compared across pseudowords, to test our hypothesis regarding decomposition of pseudowords and the processing of individual morphemes. No early or later effects were found in a temporal lobe search. Though no results were statistically significant, we found interesting results in the later processing time window that were reaching significance. At the temporal pole at 431–500 ms PSO (*p* = 0.06), we found more activity for real-root pseudowords. However, at the IFG, an area associated with world and semantic knowledge, at 315–398 ms PSO (*p* = 0.068), the reverse was true: More activity was found for nonroot pseudowords than real-root pseudowords.

## DISCUSSION

Our research attempted to shed light on the neural basis of the role of affixes in forming the meaning of words. We introduced the variable *affix semantic typicality* to reflect the fact that an affix, while often ambiguous, is associated with a predominant semantic category. In other words, within an affix’s derivational family, the semantic category with the highest type frequency is the affix’s typical meaning, such as place for “-ery” words (e.g., “bakery,” “brewery”), or person for “-ist” words (e.g., “linguist,” “artist”). Accomplishing this by using a double dissociative design of affix and meaning typicality Arabic offers us, alongside pseudowords with the affixes of interest, we found significant effects for semantic typicality in the temporal pole, as well as in the ITG/MTG area. These are areas previously implicated in lexical processing and the results are consistent with previous results on semantic composition, both at the word level (Fruchter & Marantz, 2015; Neophytou et al., 2018), and at the phrase level (Bemis & Pylkkänen, 2011; Flick et al., 2021; Li & Pylkkänen, 2021), where there is higher activity in these areas for compositions that are more coherent, plausible, and grammatical. Importantly, our results implicate semantic processing at an earlier temporal stage than previously observed. This suggests an earlier relevance of meaning, not just form, than previously supported by other studies on semantic composition of this sort. Our design allowed for a focused examination of the meaning most associated with one form, independently from the specific form itself (the affix) or the meaning itself (a place or tool, in the case of our experiment). Importantly, our study was able to assess the semantics of affixes in a novel way through Arabic. All previous psycholinguistic and neurolinguistic studies investigating affixes had designs that treated affixes as shifting meaning away from a *base meaning* provided by the root (stem), in part motivated by the ability of the root to be a free morpheme. Within Arabic, however, where the root has no fully specified base meaning and is always a bound morpheme combined with a word pattern, this conflation is eliminated.

Through a design centered on this prevalent property of Arabic, we were able to show the effect of semantic typicality in word processing. Our results indicate an interaction between form and meaning, showing that long-term top-down feedback associates certain forms with certain meanings. In other words, shared meanings among words sharing a form create an abstraction that lets us predict the expected semantic category of a given form (i.e., affix). Our results may have otherwise not supported the notion of typicality. For instance, if our hypothesis regarded not the semantic typicality of an affix, but rather its semantic *entropy*—the likelihood that a word with a certain affix has one of a set of meanings given their distribution—then our results would have reflected affix-specific variables instead of cross-affix ones. If neither entropy nor typicality were relevant in word processing, given the controlled extraneous variables within the design, we would expect a null result in the experiment for the 2 × 2 design. A trend for difference in semantic category (place vs. tool), additionally, only shows up later in processing in frontal areas. Instead, we confirm that typicality, which involves an abstraction of expected meaning, independent of specific root and affix, is relevant in the processing of words. As expected, we did not find strong behavioral effects on RT and accuracy for words contrasted in typicality. Given that this study’s question was concerned with neural substrates, the design and task were not tailored to investigate typicality through RT and accuracy as predictors. Nevertheless, through our use of pseudowords and a semantic categorization task, we elucidated that there is no evident categorization bias in the comprehension of affixes. Like Boudelaa and Marslen-Wilson’s (2015) reasoning, this may be related to an interaction with the root, since we used only low-productivity roots for real-root pseudowords, but this was aside from the focus of this study. Our behavioral results do show, however, a difference in the processing of real-root and nonroot pseudowords through RT and accuracy. The first of these were more difficult to judge in a lexical decision task, but they did not show a significant difference in their processing in the temporal lobe and only a trend toward significance in later frontal lobe areas. Moreover, null behavioral findings on semantic typicality were countered by finding an early temporal lobe effect within MEG data. Our trend of behavioral and MEG results show that these two types of data are complementary, and their conjunction and comparison is valuable for the interpretation of an experiment. In an exploratory analysis on our behavioral data, we found that defining typicality as a continuous measure of fit along a typical category yielded better models. We did not, however, extend this exploratory analysis for our MEG study since it was designed for a double dissociative analysis and not a continuous measure relationship, rendering several variables collinear where they are controlled in contrastive designs.

Another aspect of affix processing we aimed to address was the syntactic ambiguity of some affixes. We found more activity for NV ambiguous words formed with NV ambiguous affixes, across affixes, as opposed to unambiguously nominal words in the FFG at 143–250 ms PSO, and in the ITG, MTG, and STG at 131–250 ms PSO. This is consistent with previous studies that have found more activity for words with higher NV entropy (King et al., 2016: entropy correlated with anterior temporal lobe activity, peaking at 220 ms). As above, these effects are not evident behaviorally, where we mainly found frequency effects, but are found neurally. Additionally, we explored the difference in the lexicality types of our stimuli, which we divided into existing words, pseudowords with existing roots, and pseudowords with nonexistent but plausible roots. Behaviorally, we found robust differences in the RTs and accuracies of nonroot and real-root pseudowords, where responses for the latter were significantly slower and less accurate. Neurally, we found a significant difference between the processing of real words and pseudowords in all our ROIs, in the VWFA as well as the STG, MTG, and ITG areas, which further supports the full decomposition model of word processing (Rastle & Davis, 2008; Taft & Forster, 1975). We further explored the difference in processing for each of our pseudoword conditions. Although the MEG results were not significant for this comparison, we found a trending difference in the processing of real-root pseudowords versus pseudoroot pseudowords. We observed these effects nearing significance at the temporal pole (*p* = 0.06) at 431–500 mins PSO, with more activity for real root pseudowords. Additionally, we found differences in their processing in the inferior frontal cortex, with increased activity for pseudoroot pseudowords at 315–394 ms PSO (*p* = 0.068), resembling results for semantic and world knowledge violations at the phrase level in the LIFC at a similar time frame (301–330 ms; MEG: Pylkkänen et al., 2009, and fMRI: Hagoort et al., 2004). No effect, however, was found at the OFC, where previous effects of semantic well-formedness were found (Fruchter & Marantz, 2015).

Finally, we explored the difference in the processing of the different semantic categories of our design. Behaviorally, with the semantic categorization task, we found an unexpected difference in the categorization of places versus tools with a preference for the latter for categorizing new words. Neurally, since affix semantic typicality is a taxonomic relation, the spatial spread of our results (temporal lobe, MTG, and ITG) is in line with the finding that taxonomic relation processing tends to be localized in the anterior temporal lobe (Mirman et al., 2017, for a review). We also found a difference in the processing of places and tools, with higher activity for tools in BAs 41 and 42, though not in any of our primary ROIs. Given the low spatial resolution MEG offers, it is possible that this is an effect actually localized in the adjacent premotor cortex, which has been found to be sensitive to tool and action processing alongside the left middle frontal gyrus and the left IFG (Grabowski et al., 1998). Unlike results from neuropsychological and neuroimaging studies that found the left MTG to be engaged in the processing of tool words (Johnson-Frey, 2004), we did not find this result in our experiment.

Our study is not without limitations. Given that Arabic, particularly Levantine Arabic, is an under-resourced language, we had to implement different circumventions to quantify and shape our stimuli. For instance, since there is no large, annotated database for Levantine Arabic words, the words and roots were assembled manually and classified via the intuitions of the first author and through consultation with other native speakers. This is especially true for assignment of words to some of the semantic categories within our design—tools (experiment 1) and agents (experiment 2)—as well as differences in the relative use of NV ambiguous forms, a large part of which differ from modern standard Arabic, the Arabic that most language corpora are based on. Nevertheless, we prioritized the ecological validity of the word stimuli, which native speaker intuitions for well-formedness and frequency ensured. Despite the difficulties in stimulus creation and quantification, the study of under-resourced languages, especially typologically distant ones such as Arabic, is necessary, informative, and enriching to the field of morphosemantics.

### Conclusion

All in all, by investigating affixes, an understudied aspect of morphological processing, we were able to elucidate the semantic role of derivational morphemes, specifically, that their meaning typicality grants a processing advantage early in the anterior temporal lobe, as part of the ventral visual processing stream. Additionally, we replicated in Arabic findings on NV ambiguity and found interesting effects of decomposition for different kinds of pseudowords, further supporting the full decomposition model of morphological processing.

## ACKNOWLEDGMENTS

We would like to thank Jeff Walker for guidance and support during data collection, as well as Suhail Matar for valuable discussions throughout various stages of this study.

## FUNDING INFORMATION

New York University Abu Dhabi (https://dx.doi.org/10.13039/100020770), Award ID: G1001.

## AUTHOR CONTRIBUTIONS

**Marianne Azar**: Conceptualization: Lead; Formal analysis; Investigation; Methodology: Lead; Visualization; Writing – original draft: Lead; Writing – review & editing: Lead. **Alec Marantz**: Conceptualization: Supporting; Funding acquisition; Methodology: Supporting; Writing – original draft: Supporting; Writing – review & editing: Supporting.

## CODE AND DATA AVAILABILITY STATEMENT

The code and data are publicly available at https://osf.io/k6tca/.

## Supplementary Material



## TECHNICAL TERMS

Morpheme: Any of the smallest meaningful units within a language. They are divided into roots and affixes, and they are either free (i.e., can stand on their own) or bound (need to be attached to another morpheme to form a word). In the word “unlockable” there are three morphemes: two bound affixes (“un-“ and “-able”) and one free root (“lock”). Morphemes contain semantic and/or syntactic information.
Root-and-pattern Arabic morphology: The currently most supported account of Arabic morphology and morphological processing. Under this account, Arabic morphology is characterized by nonconcatenative morphemes tthat combine to form a word. Every word is minimally composed by a root and a pattern. For instance, in the word “maktab,” the root is KTB, and the pattern is ma _ _a _. The root is predominantly a three-consonant string which is semantically polysemous in the words it occurs in and is syntactically underspecified. The pattern is a prosodic structure with vowels and often consonants wherein the root is interleaved in determined positions of each specific pattern, and it provides semantic and syntactic information, though polysemous or ambiguous, respectively, in isolation.
Double dissociation: A paradigm commonly used in neuropsychology to establish a dissociation between two variables sharing similar features. For example, establishing an independence between A and B would require two instances where instance 1 has A not B, and instance 2 has B not A. Without one of these two instances, it is difficult to establish that either is not dependent on the other. If only instances of A not B are found, it is possible that B is reliant on A.
Magnetoencephalography (MEG): A neuroimaging technique which passively records the magnetic potential produced by electric currents emitted by the brain using highly sensitive magnetometers. Although it has a coarser spatial resolution than the more widely known fMRI technique, MEG is distinct in its high temporal resolution, which can be as fine as 2 ms. For this reason, it is a technique that is highly useful for studying cognitive processes wherein rapidly unfolding temporal neural dynamics are of interest. Examples of this include language and music processing.
Visual word form area (VWFA): An area in the left occipitotemporal region (fusiform gyrus) associated with visual word form processing. Although its spatial specification varies within the discipline, it has robustly been found to respond to the processing of word forms—and not, e.g., symbol strings—and is sensitive to the orthography of a language. It has occasionally been associated with lexical processing.
M170: An MEG response peak between 150 and 200 ms post-word onset characteristically in the left inferior occipitotemporal area (specifically the fusiform area) that is sensitive to morphological complexity of words.
Lexical decision task: A common experimental paradigm used in psycholinguistics in which participants are asked to classify stimuli into words and nonwords. Common outcome variables are reaction time and accuracy as well as brain responses in relevant brain regions depending on the aspect of language being investigated (e.g., syntactic composition or pragmatic processing).
