# Supplementary material for: Semantic Typicality of Affixes Facilitates Word Processing: MEG Evidence From Arabic
Source: Neurobiol Lang (Camb). 2025 Dec 1;6:NOL.a.24. doi: 10.1162/NOL.a.24 (PMC12674548; doi:10.1162/NOL.a.24)
Supplement: Supplementary file 1 [file nol-6-1-24-s001.pdf]

SUPPLEMENTARY MATERIAL

2. Introduction

2.3. Arabic derivational affixes: necessary and informative

Example sentences with words sharing a root. Highlighted words are thematically related			
Example 1			
كتب	الكاتب	مكتوب	مكتبه في
**K**a**t**a**b**a	El-**kateb**	ma**ktūb**	Fi ma**kt**a**b**ihi
Wrote	The writer	letter	in office-his
The **writer wrote** a **letter** in his **office.**			
Example 2			
حكم	الحاكم	على المحكوم	في المحكمة
hakama	El-hakem	Ala el-mahkūm	Fi el-ma**hk**a**ma**
Sentenced	The-ruler	On the-convict	in the-court
The **ruler sentenced** the **convict** in **court.**			

Table 4A*: Shared roots with a thematic relation*

**_Example sentences with words sharing an affix. Highlighted words are taxonomically related._**								
Example 1: typical meaning (places)								
في وسط	المدينة	هناك	مسرح،	مسجد،	مصرف،	ملعب،	ومقهى	تاريخي
Fi wasat	el-madina	hunak	**Ma**sra**h**,	**Ma**sj**e**d,	**Ma**sr**e**f,	**Ma**la**a**b,	wa **ma**qha	Tari:khiy
In center	The-city	there	theater,	mosque,	bank,	stadium,	and café	historical
In the city center there is a **theater**, a **mosque**, a **bank**, a **stadium**, and a historical **café**.								
Example 2: atypical meanings (concepts, tools)								
بالمبدأ	ستجد	الملقط،	المبرد،	والمقص	في	خزانة	الحمام	
Bel-**ma**bd**a**ʔ	sa-tajed	el-**ma**lq**a**t,	el-**ma**br**a**d,	wal-**m**q**a**ss	fi	khiza:nat	al-himmam	
In principle	will-find[2SgM]	the-tweezers	The-nail file	And the-scissors	in	cabinet	the-bathroom	
In **principle**, you will find the **tweezers**, **nail file**, and **scissors** in the bathroom cabinet.								

Table 4B: *Shared affixes with a taxonomic relation, with a categorical distinction of affix category.*

4. Results

4.1. Behavioral results

Lexicality:

Residuals Summary

**Min**	**1Q**	**Median**	**3Q**	**Max**
-0.8704	-0.3188	-0.1380	0.1797	2.2005

**Predictor**		**Estimate**	**SE**	**t**	**p**
	(Intercept)	0.281	0.019	14.468	<.001 ***
Lexicality	**NW**	-0.167	0.023	-7.108	<.001 ***
	**NR**	-0.284	0.024	-11.760	<.001 ***
Affix	**B**	-0.025	0.014	-1.792	0.073
	**C**	-0.128	0.017	-7.468	<.001 ***
	**D**	-0.004	0.017	-0.271	0.787
	**E**	-0.163	0.015	-10.804	<.001 ***

	Frequency		-0.082	0.004	-20.477	<.001 ***
*Lexicality *Affix*	NW	B	-0.013	0.024	-0.566	0.571
		C	-0.084	0.028	-2.992	0.00278 **
		D	0.012	0.029	0.429	0.668
		E	0.018	0.024	0.753	0.452
	NR	B	-0.010	0.025	-0.425	0.671
		C	-0.063	0.026	-2.384	0.01713 *
		D	-0.004	0.026	-0.134	0.893
		E	0.014	0.025	0.546	0.585

Residuals Summary

Min	1Q	Median	3Q	Max
-7.0271	0.2003	0.3077	0.4967	1.4632

**Random Effects**						
	**Variance**		**SD**	**N Observations**		
Participant	0.1833		0.4281	19334		
**Predictor**			**Estimate**	**SE**	**z**	**p**
	**(Intercept)**		1.24989	0.09392	13.308	<.001 ***
Lexicality	**NW**		0.951	0.0777	-12.224	<.001 ***
	**NR**		1.455	0.133	10.921	<.001 ***
Affix	**B**		0.13622	0.072	1.889	0.058
	**C**		1.598	0.13	12.309	<.001 ***
	**D**		1.093	0.108	10.088	0.787
	**E**		0.13654	0.079	1.737	0.0823
Lexicality *Affix	**NW**	**B**	0.302	0.113	2.678	0.0074 **
		**C**	-0.235	0.175	-1.338	0.1809
		**D**	-0.767	0.15	-5.105	<.001***
		**E**	0.541	0.118	4.568	<.001***
	**NR**	**B**	-0.036	0.191	-0.188	0.851
		**C**	-1.529	0.218	-7.013	<.001***
		**D**	-0.953	0.208	-4.57	<.001***
		**E**	0.361	0.209	1.72	0.0855

Table 11 (A and B) *Linear (A) and logistic (B) regression model summaries of lexicality, affix, and frequency predicting RT (A) and accuracy (B), respectively.*

Semantic Typicality:

Residuals Summary

Min	1Q	Median	3Q	Max
-1.611	-0.517	-0.231	0.284	3.617

Predictor		Estimate	SE	t	p
	(Intercept)	2.271	0.056	40.549	<.001 ***
Typicality	**Atypical**	-0.103	0.081	-1.266	0.205
Affix	**B**	-0.127	0.032	-3.957	<.001 ***
**Frequency**		-0.154	0.012	-12.593	<.001 ***
Typicality *Affix	**Atypical B**	0.178	0.048	3.744	<.001 ***
Typicality * Frequency	**Atypical**	-0.017	0.018	-0.956	0.339

Residuals Summary

Min	1Q	Median	3Q	Max
-11.934	0.122	0.253	0.463	4.426

**Random Effects**					
	**Variance**	**SD**	**N Observations**		
**Participant**	0.5	0.707	4674		
	**Predictor**	**Estimate**	**SE**	**z**	**p**
	**(Intercept)**	-2.400	0.229	-10.492	<.001 ***
Typicality	**Atypical**	0.132	0.269	0.489	0.625
Affix	**B**	-0.077	0.113	-0.678	0.498
Frequency		0.945	0.049	19.148	<.001 ***
*Typicality * Affix*	**Atypical B**	-0.157	0.169	-0.927	0.354
*Typicality * Frequency*	**Atypical**	0.116	0.074	1.565	0.118

Table 12 (A and B)*: Linear (A) and logistic (B) regression model summaries of typicality, affix, and frequency predicting RT (A) and accuracy (B), respectively.*

Exploratory analysis: continuous semantic category fit:

Residuals Summary

Min	1Q	Median	3Q	Max
-1.5403	-0.515	-0.2251	0.284	3.67

Predictor		Estimate	SE	t	p
	(Intercept)	2.382	0.122	19.570	<.001 ***
Place fit		-0.369	0.090	-4.112	<.001 ***

Tool fit		0.018	0.142	0.123	0.902
Affix	**B**	-0.058	0.146	-0.39	0.696
**Frequency**		-0.156	0.010	-16.148	<.001 ***
Place fit *Affix	**B**	0.209	0.108	1.931	0.054
Tool fit * Affix		-0.153	0.173	-0.887	0.375

**Residuals Summary**				
**Min**	**1Q**	**Median**	**3Q**	**Max**
-12.5634	0.107	-0.237	0.458	3.95

**Random Effects**					
	**Variance**	**SD**	**N Observations**		
**Participant**	0.518	0.720	4656		
	**Predictor**	**Estimate**	**SE**	**z**	**p**
	**(Intercept)**	-2.87910	0.42572	-6.763	<.001***
Place fit		1.12947	0.33193	3.403	<.001***
Tool fit		-0.097	0.507	-0.191	0.848
Affix	**B**	-1.267	0.501	-2.529	0.011*
**Frequency**		1.006	0.043	23.492	<.001***
Place fit *Affix	**B**	-0.307	0.402	-0.763	0.445
Tool fit * Affix	**B**	2.403	0.631	3.811	<.001***

Table 13 (A and B)*: Linear (A) and logistic (B) regression model summaries of place fit, tool fit, affix, and frequency predicting RT (A) and accuracy (B), respectively.*

Syntactic Ambiguity:

**Residuals Summary**				
**Min**	**1Q**	**Median**	**3Q**	**Max**
-1.109	-0.459	-0.224	0.203	3.817

**Predictor**		**Estimate**	**SE**	**t**	**p**
	(Intercept)	1.766	0.042	42.159	<.001 ***
Syntactic Ambiguity	**NV**	0.028	0.030	0.934	0.35
Affix	**C**	-	-	-	-
	**D**	0.231	0.029	7.830	<.001 ***
Frequency		-0.118	0.010	-11.717	<.001 ***

Residuals Summary

Min	1Q	Median	3Q	Max
-10.428	0.124	0.203	0.358	2.238

**Random Effects**					
	**Variance**	**SD**	**N Observations**		
**Participant**	0.583	0.764	4407		
	**Predictor**	**Estimate**	**SE**	**z**	**p**
	**(Intercept)**	-1.7896	0.221	-8.102	<.001 ***
Syntactic Ambiguity	**NV**	0.2044	0.158	1.298	0.194
Affix	**C**	-	-	-	-
	**D**	0.1365	0.165	0.830	0.407
Frequency		0.9602	0.052	18.536	<.001 ***

Table 14 (A and B): *Linear (A) and logistic (B) regression model summaries of syntactic ambiguity, affix, and frequency predicting RT (A) and accuracy (B), respectively.*
